# Supplementary material for: Predictors of quality of life in individuals with non-traumatic unilateral transtibial amputation
Source: PLoS One. 2025 Jul 14;20(7):e0327781. doi: 10.1371/journal.pone.0327781 (PMC12258552; doi:10.1371/journal.pone.0327781)
Supplement: Attachment — Submitted filename: Response to the reviewers.docx [file pone.0327781.s003.docx]

**Journal Requirements:**

1.Please ensure that your manuscript meets PLOS ONE's style requirements, including those for file naming. The PLOS ONE style templates can be found at

<https://journals.plos.org/plosone/s/file?id=wjVg/PLOSOne_formatting_sample_main_body.pdf> and

[https://journals.plos.org/plosone/s/file?id=ba62/PLOSOne_formatting_sample_title_authors_affiliations.pdf](https://track.editorialmanager.com/CL0/https:%2F%2Fjournals.plos.org%2Fplosone%2Fs%2Ffile%3Fid=ba62%2FPLOSOne_formatting_sample_title_authors_affiliations.pdf/1/010f0196401efdfb-4f2293a1-6db6-46a9-a82b-82e70a4dec96-000000/wA7esOWbKI61w6VFu3XAxrRisi05iS-2B0t8L_qr32Q=207)

**Response:** We checked the PLOS ONE's style requirements and the required changes have been made in the text.

2. We noticed you have some minor occurrence of overlapping text with the following previous publication(s), which needs to be addressed:

<https://journals.lww.com/poijournal/abstract/9900/prosthesis_and_health_related_factors_of_mobility.304.aspx>

In your revision ensure you cite all your sources (including your own works), and quote or rephrase any duplicated text outside the methods section. Further consideration is dependent on these concerns being addressed.

**Response**: Thank you for your comment. In this revised version of the manuscript, we have added a sentence to the discussion that highlights the findings of our previous article, along with the corresponding citation. Additionally, we have rephrased the overlapping text to eliminate any duplicated phrases. These changes have been made using track changes.

3. We note that your Data Availability Statement is currently as follows: All relevant data are within the manuscript and in Supporting Information files.

Please confirm at this time whether or not your submission contains all raw data required to replicate the results of your study. Authors must share the “minimal data set” for their submission. PLOS defines the minimal data set to consist of the data required to replicate all study findings reported in the article, as well as related metadata and methods (<https://journals.plos.org/plosone/s/data-availability#loc-minimal-data-set-definition>).

For example, authors should submit the following data:

- The values behind the means, standard deviations and other measures reported;

- The values used to build graphs;

- The points extracted from images for analysis.

Authors do not need to submit their entire data set if only a portion of the data was used in the reported study.

If your submission does not contain these data, please either upload them as Supporting Information files or deposit them to a stable, public repository and provide us with the relevant URLs, DOIs, or accession numbers. For a list of recommended repositories, please see <https://journals.plos.org/plosone/s/recommended-repositories>.

If there are ethical or legal restrictions on sharing a de-identified data set, please explain them in detail (e.g., data contain potentially sensitive information, data are owned by a third-party organization, etc.) and who has imposed them (e.g., an ethics committee). Please also provide contact information for a data access committee, ethics committee, or other institutional body to which data requests may be sent. If data are owned by a third party, please indicate how others may request data access.

**Response:** The anonymized scores from the questionnaire scale have been submitted as a supporting information file.

4. Please review your reference list to ensure that it is complete and correct. If you have cited papers that have been retracted, please include the rationale for doing so in the manuscript text, or remove these references and replace them with relevant current references. Any changes to the reference list should be mentioned in the rebuttal letter that accompanies your revised manuscript. If you need to cite a retracted article, indicate the article’s retracted status in the References list and also include a citation and full reference for the retraction notice.

**Response:** We used the Retraction Watch Database ([https://retractionwatch.com](https://retractionwatch.com/)) to search the reference list for any retractions. However, we did not identify any retracted articles. If you come across any retracted articles cited in this study, please let us know.

**Reviewers' comments:**

**Reviewer #1:**  I would like to start by thinking the authors for their work in this area. It is always important to learn about all the factors that impact quality of life for our patients with amputations. I feel that I have only minor suggestions, which I will detail below.

**Response:** Thank you for your thorough review of our article. Your constructive comments were invaluable and greatly appreciated. We believe that your insights have significantly strengthened our work.

**Comment:** Line 77-78: The authors have a sentence starting with "Studies have indicated..." This should have a reference.

**Response:** We have now cited appropriate references for each sentence individually within the paragraph to improve clarity and attribution. These changes are highlighted in yellow in line 81 of the revised manuscript.

**Comment:** Line 105-107: The authors indicate that the focus of the study is to identify modifiable factors to enhance prosthesis rehabilitation. This is actually not what is done. They are looking at prosthesis parameters that correlate with high QoL. This may include modifications that can be implemented into the prosthesis but modifiable "factors" to enhance prosthesis rehabilitation would include modification to disease management like diabetes control as well as interventions focused on items like falls confidence, body image, mobility and balance training, community access and integration, access to education and peer support, etc. So I guess I am saying, try not to be too broad with these statements.

**Response:** Thank you for your thoughtful comment. As you correctly pointed out, the primary focus of our study is to examine how prosthetic fitting and components relate to quality of life, rather than broadly identifying all modifiable factors involved in prosthetic rehabilitation. We have revised the sentence to more accurately reflect the scope of the study. These changes can be found in lines 105–108 of the revised manuscript and are highlighted in yellow.

**Comment:** Discussion line 206: "Our regression analysis results revealed that the comfort subscale of CLASS is the strongest predictor of quality of life in people with non-traumatic LLA." I suggest being more specific to the study as it was in people with non-traumatic, unilateral, transtibial amputations, when looking only at prosthetic related parameters.

**Response:** Thank you for your attention. We have revised the sentence to better reflect our findings. The updated sentence can be found in lines 220-221 of the revised manuscript and is highlighted in yellow. Additionally, this change has been applied throughout the manuscript and is also highlighted in yellow.

**Comment:** line 218: needs a reference

**Response:** Thank you for your comment. The required reference has been added, and the change is highlighted in yellow in line 234 of the revised manuscript.

**Comment:** Line 221-223: There is a big assumption in the sentence "Therefore, it can be concluded that prosthetic socket comfort will increase the social interaction of ..." I recommend tempering this a bit by saying for example, ... it can be concluded that prosthetic socket comfort may increase their willingness to take part in social interactions...

**Response:** Thank you for your valuable suggestion. We have revised the sentence. The change is highlighted in yellow in lines 241–243 of the revised manuscript.

**Comment:** Line 221-226: repetitive statements across a few sentences.

**Response:** Thank you for your helpful feedback. As you suggested, we revised and condensed the original repetitive sentences into a more concise statement, which has been highlighted in yellow in lines 234–241 of the revised manuscript.

**Comment:** Line 262-278: Interesting discussion but the classification of articulated vs. non is unfortunately not helpful. For example, an energy storage foot is not articulated but can mimic three rockers and provide very good biomechanics in both swing and stance phase. so to lump this with a SACH would not help the reader to understand the impact of foot type. I would suggest, if able to subdivide to SACH, single axis, multi axial and Energy storage and see if there is a correlation. (if able). If not, this may be something for further study.

**Response:** Thank you for highlighting this important point. We totally agree that the function of a SACH foot is different with energy storing ones. Therefore, we have mentioned this important point in the limitation section for future studies. Please see lines 327-335.

**Comment:** line 307: sentence ending with "... non-traumatic LLA." I would recommend correcting to ...non-traumatic, unilateral, transtibial amputations. This is an important distinction as the authors, form this study cannot generalize to bilateral amputees or transfemoral amputees or above.

**Response:** Thank you for your valuable suggestion. We have revised the sentence. The change is highlighted in yellow in lines 340-341 of the revised manuscript.

**Reviewer #2:** Thank you for your thorough review of our article. Your constructive comments were invaluable and greatly appreciated. We believe that your insights have significantly strengthened our work.

**Comment:** In the discussion, the role of appearance could be explored further, as the study’s findings (weak association) are only partially in line with the cited literature.

**Response:** Thank you for your comment. We have added further explanation to the discussion to clarify the weak association between satisfaction with socket appearance and quality of life in our study. The updated paragraph is highlighted in yellow in lines 258-263 of the discussion section.

**Comment:** the section regarding the length of the residual limb could be expanded, as this is an important factor in the literature on this topic: Have other studies highlighted that the length of the residual limb does not affect quality of life?

**Response:** Thank you for your insightful comment. We recognize that the original paragraph may have caused some confusion regarding the source of the findings. To clarify, the discussion in this section reflects the results of our own study rather than findings from previous literature. Specifically, our study did not identify a significant correlation between residual limb length and quality of life. To the best of our knowledge, there is limited literature directly examining the relationship between residual limb length and quality of life, particularly in individuals with non-traumatic lower-limb amputation. While many studies emphasize the biomechanical and functional implications of residual limb length (including socket fit, energy expenditure, and prosthetic alignment) we were unable to locate studies that explicitly confirm or refute its influence on quality of life. Therefore, we attempted to interpret our findings within the context of known biomechanical principles and the uneven distribution of participants across residual limb length categories. We have revised the paragraph accordingly to ensure this distinction is clear. The change is highlighted in yellow in lines 298-301 of the revised manuscript.
